# Department of Defense influenza and other respiratory disease surveillance during the 2009 pandemic

**DOI:** 10.1186/1471-2458-11-S2-S6

**Published:** 2011-03-04

**Authors:** Ronald L Burke, Kelly G Vest, Angelia A Eick, Jose L Sanchez, Matthew C Johns, Julie A Pavlin, Richard G Jarman, Jerry L Mothershead, Miguel Quintana, Thomas Palys, Michael J Cooper, Jian Guan, David Schnabel, John Waitumbi, Alisa Wilma, Candelaria Daniels, Matthew L Brown, Steven Tobias, Matthew R Kasper, Maya Williams, Jeffrey A Tjaden, Buhari Oyofo, Timothy Styles, Patrick J Blair, Anthony Hawksworth, Joel M Montgomery, Hugo Razuri, Alberto Laguna-Torres, Randal J Schoepp, David A Norwood, Victor H MacIntosh, Thomas Gibbons, Gregory C Gray, David L Blazes, Kevin L Russell, AFHSC-GEIS Influenza Surveillance Writing Group

**Affiliations:** 1 Armed Forces Health Surveillance Center, 503 Robert Grant Avenue, Silver Spring, MD 20910, USA; 2 Armed Forces Research Institute of Medical Sciences, 315/6 Rajavithi Road, Bangkok, Thailand 10400; 3 Center for Disaster and Humanitarian Assistance Medicine, Uniformed Services University of the Health Sciences, F. Edward Hébert School of Medicine, 4301 Jones Bridge Road, Bethesda, MD 20814, USA; 4 Public Health Region-South, Building 2472, Schofield Road, Fort Sam Houston, TX 78234, USA; 5 Landstuhl Regional Medical Center, Department of Pathology and Area Laboratory Services, CMR 402, APO AE 09180, USA; 6 Public Health Region-Europe, CMR 402, APO AE 09180, USA; 7 Public Health Region-Pacific, Unit 45006, APO AE 96343, USA; 8 U.S. Embassy, Attention: MRU, United Nations Avenue, Post Office Box 606, Village Market 00621 Nairobi, Kenya; 9 Department of Defense Veterinary Food Analysis & Diagnostic Laboratory, 2472 Schofield Road, Suite 2630, Fort Sam Houston, TX 78234, USA; 10 USAMEDDAC-Korea, Microbiology Section, Unit 15244, Box 459, APO AP 96205, USA; 11 Naval Medical Research Unit Number 2, Kompleks Pergudangan DEPKES R.I., JI. Percetakan Negara II No. 23, Jakarta 10560, Indonesia; 12 Naval Medical Research Unit No. 3, Extension of Ramses Street, Adjacent to Abbassia Fever Hospital, Postal Code 11517, Cairo, Egypt; 13 U.S. Navy Environmental Preventive Medicine Unit No. 2, 1887 Powhatan Street, Norfolk, VA 23511, USA; 14 Naval Health Research Center, 140 Sylvester Road, San Diego, CA 92106, USA; 15 Naval Medical Research Center Detachment, Centro Medico Naval “CMST,” Av. Venezuela CDRA 36, Callao 2, Lima, Peru; 16 U.S. Army Medical Research Institute of Infectious Diseases, Diagnostic Systems Division, 1425 Porter Street, Fort Detrick, MD 21702, USA; 17 U.S. Air Force School of Aerospace Medicine, 2513 Kennedy Circle, Building 180, Brooks City Base, TX 78235, USA; 18 Department of Environmental and Global Health, College of Public Health and Health Professions, University of Florida, Post Office Box 100188, Gainesville, FL 32610, USA; 19 Walter Reed Army Institute of Research, Emerging Infectious Diseases Research Unit, 503 Robert Grant Avenue, Silver Spring, MD 20910, USA; 20 Australian Army Malaria Institute, Gallipoli Barracks, Enoggera, QLD 4051, Australia; 21 Johns Hopkins University Applied Physics Laboratory, 11100 Johns Hopkins Road, Laurel, MD 20723, USA; 22 U.S. Navy and Marine Corps Public Health Center, 620 John Paul Jones Circle, Suite 1100, Portsmouth, VA 23708, USA; 23 Laboratory for Emerging Infectious Diseases, University of Buea, Post Office Box 63, Buea, Cameroon; 24 Global Viral Forecasting Initiative, 1 Sutter, Suite 600, San Francisco, CA 94104, USA

## Abstract

The Armed Forces Health Surveillance Center’s Division of Global Emerging Infections Surveillance and Response System (AFHSC-GEIS) supports and oversees surveillance for emerging infectious diseases, including respiratory diseases, of importance to the U.S. Department of Defense (DoD). AFHSC-GEIS accomplishes this mission by providing funding and oversight to a global network of partners for respiratory disease surveillance. This report details the system’s surveillance activities during 2009, with a focus on efforts in responding to the novel H1N1 Influenza A (A/H1N1) pandemic and contributions to global public health. Active surveillance networks established by AFHSC-GEIS partners resulted in the initial detection of novel A/H1N1 influenza in the U.S. and several other countries, and viruses isolated from these activities were used as seed strains for the 2009 pandemic influenza vaccine. Partners also provided diagnostic laboratory training and capacity building to host nations to assist with the novel A/H1N1 pandemic global response, adapted a Food and Drug Administration-approved assay for use on a ruggedized polymerase chain reaction platform for diagnosing novel A/H1N1 in remote settings, and provided estimates of seasonal vaccine effectiveness against novel A/H1N1 illness. Regular reporting of the system’s worldwide surveillance findings to the global public health community enabled leaders to make informed decisions on disease mitigation measures and controls for the 2009 A/H1N1 influenza pandemic. AFHSC-GEIS’s support of a global network contributes to DoD’s force health protection, while supporting global public health.

## Background

In response to the 1996 Presidential Directive (NSTC-7), the U.S. Department of Defense (DoD) established the Global Emerging Infections Surveillance and Response System (DoD-GEIS) in 1997, with the mission to monitor newly emerging and re-emerging infectious diseases (EIDs) among U.S. servicemembers and dependent populations [1]. Comparable to their global burden of disease, respiratory infections are responsible for 25 percent to 30 percent of both outpatient illness and hospitalizations among U.S. military personnel [2,3]. Influenza and adenovirus infections are among the etiologies that greatly contribute to morbidity and mortality in military members [4]. During the 1918 influenza pandemic, the U.S. military experienced attack rates as high as 25 percent and case fatality rates averaging 5 percent (ranging from 1 percent to 8 percent) [5].

**Figure 1:**
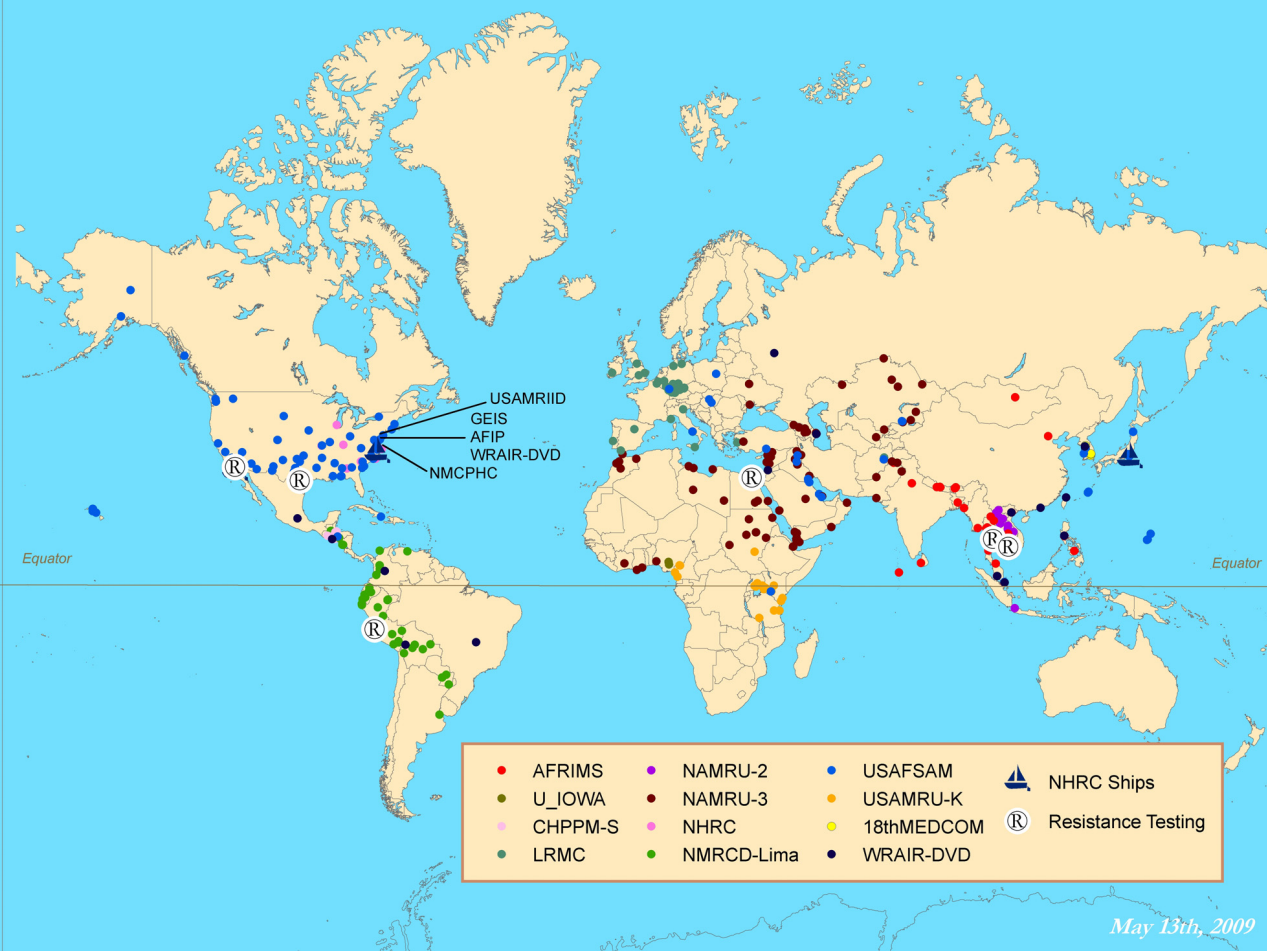
AFHSC-GEIS global influenza surveillance presence worldwide, as of May 2009.

DoD-GEIS, a division of the Armed Forces Health Surveillance Center (AFHSC) since early 2008, centralized the coordination of DoD influenza and other respiratory disease surveillance efforts beginning in 1998. The program was expanded with 2006 congressional supplementary appropriations [6-8]. Subsequent funding in 2007-2009 has maintained this effort. Today, AFHSC-GEIS provides direction, funding and oversight to a system that consists of a network of global partners, including approximately 500 sites in 70 countries (Figure 1).

During the past four years (2006-2009), the AFHSC-GEIS influenza surveillance system increased support for avian and pandemic influenza preparedness to include activities in surveillance and response to newly identified strains and pandemics, such as H5N1 and the 2009 novel A/H1N1. By supporting global surveillance and directing response efforts, DoD serves as a sentinel for local epidemics and can assist in limiting disease transmission. An immediate focus of DoD is decreasing the impact of the novel A/H1N1 pandemic on the armed forces, including reducing recruit- and other training-associated illnesses and deaths, and controlling secondary viral and bacterial associated morbidity. These efforts are similar in intent to those undertaken at the time of the appearance of the new virus strain during the 1918 H1N1 pandemic when efforts were also made to reduce the impact of the virus on the military during World War I.

The AFHSC-GEIS influenza surveillance system plays a major role in the U.S. government’s (USG) contributions to the global surveillance of influenza viruses and contributes to the World Health Organization’s (WHO) Global Influenza Surveillance Network [9]. Core components of the AFHSC-GEIS influenza surveillance system are a network of specialized diagnostic and reference laboratories in the continental United States, medical treatment facilities within the Military Health System, and five DoD overseas laboratories, along with their respective detachments. The system, built around networks of hub and satellite laboratories, comprises many joint ventures with host countries.

This article focuses on the 2009 activities and accomplishments of the AFHSC-GEIS laboratory-based network regarding global surveillance for respiratory diseases and responding to the novel A/H1N1 influenza pandemic. These activities are described relative to, and by means of, AFHSC-GEIS strategic goals: surveillance and response; training and capacity building; research, innovation and integration; and assessment and communication of value added.

## 2009 contributions

### Surveillance and response

During April 2009, the first two U.S. cases of novel A/H1N1 were detected in two separate Naval Health Research Center (NHRC) surveillance projects supported by AFHSC-GEIS. In the first instance, NHRC investigators collected a specimen from a 10-year-old DoD dependent who had enrolled in a biomedical trial to test a new influenza diagnostic platform conducted at the Naval Medical Center, San Diego, Calif. Initial results by an external reference laboratory suggested an influenza A/untypable virus [10,11]. At the same time, a 9-year-old female from the U.S./Mexico border was sampled in a collaborative surveillance study with the Centers for Disease Control and Prevention’s (CDC) Border Infectious Disease Surveillance Project. NHRC determined infection from an influenza A/untyped virus. Further testing on the IBIS T5000 platform, which infers H and N types from multiple genomic signatures, indicated an influenza A/swine/H1 virus. Samples from both patients were shipped to the CDC for confirmation and characterization.

Shortly thereafter, the U.S. Air Force School of Aerospace Medicine (USAFSAM) detected two near simultaneous cases among military dependents in the San Antonio area. The WHO used three of the strains (A/California/7/2009, A/California/4/2009 and A/Texas/5/2009) as potential strains for the 2009 pandemic influenza vaccine. A/California/7/2009 was eventually selected as the seed strain [9].

In addition to detecting several of the initial cases of novel A/H1N1 within the U.S., AFHSC-GEIS partner laboratories were instrumental in monitoring the global spread of the virus. The Armed Forces Research Institute of the Medical Sciences (AFRIMS) laboratory was the first to detect novel A/H1N1 virus in Nepal and Bhutan, while the Naval Medical Research Unit No. 2 (NAMRU-2) provided support for the initial confirmation on novel A/H1N1 in Cambodia and Lao People’s Democratic Republic. The U.S. Army Medical Research Unit-Kenya (USAMRU-K), another AFHSC-GEIS partner, supported initial laboratory confirmation for Kenya and the Republic of Seychelles, and the Naval Medical Research Center Detachment (NMRCD) identified the first cases in Peru and supported initial confirmation in Colombia and Ecuador. Additionally, NHRC diagnosed the first infection in Guam/Micronesia, and the Naval Medical Research Unit No. 3 (NAMRU-3) not only identified the first cases in Kuwait, but also confirmed outbreaks in Afghanistan, Bahrain, Djibouti, Egypt and Lebanon.

Results of AFHSC-GEIS-sponsored influenza surveillance sample testing were reported via host-nation collaborators to their respective ministries to ensure the ministries could make informed and timely decisions about influenza control. The AFHSC-GEIS network surveillance support of the 2009 influenza pandemic was instrumental in the timely tracking and monitoring of the virus. In recognition of AFHSC-GEIS support, the AFRIMS field laboratory in Cebu was made an official Philippine Department of Health testing laboratory, Public Health Command Region-South (PHCR-South) assisted the Guatemalan Ministry of Health Influenza laboratory in becoming a National Influenza Center (NIC), and the Peruvian Instituto Nacional de Salud (National Institute of Health) awarded a commendation medal to NMRCD for its support in the pandemic response.

In addition to the novel A/H1N1 pandemic support provided to foreign host nations, AFHSC-GEIS network partners continued to support influenza and respiratory disease surveillance among military recruits, active-duty servicemembers, and U.S. military beneficiaries. AFHSC-GEIS supported the timely surveillance and rapid diagnosis of circulating influenza and other respiratory viruses within our overseas military populations through its partners in Europe (PHCR-Europe and Landstuhl Regional Medical Center (LRMC)), Japan (PHCR-Pacific and Naval Hospital Yokosuka), and the Republic of Korea (Brian Allgood Army Community Hospital (BAACH)).

Although many network laboratories, such as LRMC and BAACH, could not confirm a novel A/H1N1 infection during the initial months of the pandemic, the facilities provided a strong presumptive diagnosis of novel A/H1N1 based on their findings of untypable influenza A infections. With AFHSC-GEIS support, BAACH became one of the first U.S. overseas laboratories capable of providing on-site diagnosis of novel A/H1N1 for U.S. military personnel and their families. Likewise, the ability of the Navy Environmental Preventive Medicine Unit No. 2, in collaboration with NAMRU-3, to stand up a novel A/H1N1 testing site in Kuwait on short notice also helped provide a timely diagnosis for deployed clinicians. In turn, this effort helped ease tensions between the U.S. military and host countries in Southwest Asia and the Middle East by allowing the rapid identification and subsequent isolation of infected individuals to reduce the likelihood of transmitting influenza virus to local civilians.

A significant challenge for AFHSC-GEIS partners in 2009 was the need to balance their novel A/H1N1 pandemic response with their ability to continue surveillance efforts for other influenza viruses and respiratory diseases (e.g., adenovirus), including potential zoonotic viruses. Over 50 percent of EIDs are zoonotic, including the H5N1 and 2009 novel A/H1N1 viruses [12]. In 2009, AFRIMS and NAMRU-2 scientists, in collaboration with the University of Iowa’s Center for Emerging Infectious Diseases, further strengthened important research of the human-animal interface and epidemiology of influenza viruses by expanding established cohort-based studies and creating new ones in five countries (Cambodia, Mongolia, Nigeria, Romania and Thailand). The endeavor allowed researchers to examine risk factors and transmission patterns of influenza at the human-animal interface.

**Figure 2:**
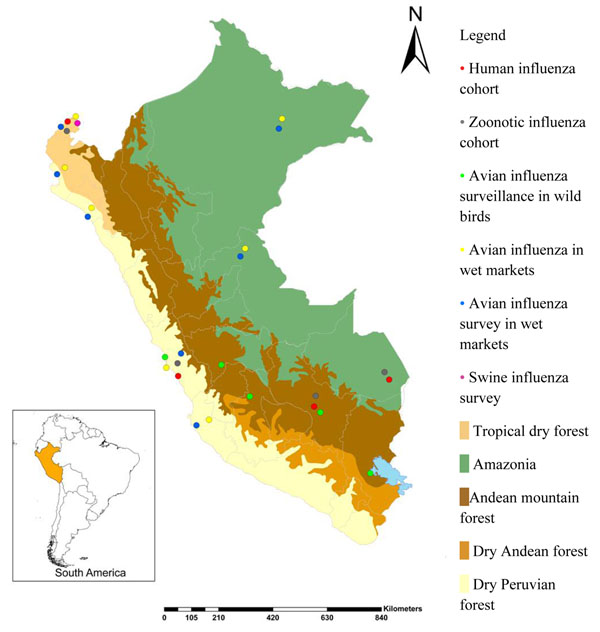
Naval Medical Research Center Detachment influenza surveillance activities in Peru during 2009.

Likewise, USAMRU-K initiated similar surveillance work in Uganda and continued to conduct migratory bird surveillance to monitor and track the spread of highly pathogenic avian influenza (HPAI). NMRCD conducted similar migratory bird surveillance in Peru. In conjunction with the CDC, NAMRU-3 and NMRCD initiated population-based, influenza-like illness and severe acute respiratory illness surveillance efforts among hospital and community cohorts in Egypt and Peru, respectively (Figure 2).

Finally, AFHSC-GEIS partners provided laboratory diagnosis for 37 of the 52 (71 percent) reported cases of human H5N1 infection worldwide in 2009 [3]. NAMRU-3, serving as the WHO’s Eastern Mediterranean Regional Influenza Reference Center (EMRO), provided laboratory diagnosis for 36 of 38 (95 percent) of reported cases of human H5N1 infection in Egypt, and NAMRU-2 identified an additional case in Cambodia [13].

## Training and capacity building

Although these initiatives are more fully addressed in other articles within this supplement, the strategic goal of training and capacity building was a significant focus of AFHSC-GEIS influenza funding [14,15]. Nearly all AFHSC-GEIS partners assisted with training and capacity building programs. While the primary objective of these programs is to develop and strengthen global surveillance capacity, the endeavors have the added benefit of improving USG civil-military and military-military relations with host nations. AFHSC-GEIS provided funding to the Center for Disaster and Humanitarian Assistance Medicine at the Uniformed Services University to conduct 14 training sessions for 36 countries. A total of 885 individuals attended the training in support of U.S. Combatant Command partnerships with priority nations.

AFHSC-GEIS funding also supported the Kenyan NIC designation in late 2009 as the WHO East Africa regional influenza laboratory through the assistance of USAMRU-K. Within three months of the novel A/H1N1 virus introduction into the region, the Kenyan NIC received and tested over 1,500 influenza samples from Kenya, Republic of Seychelles and Somalia (Additional File 1). While serving as the WHO’s EMRO reference laboratory, NAMRU-3 supported the development of NICs in Afghanistan, Iraq and Jordan, and the sustainment of NICs in Egypt, Kuwait, Oman, Pakistan, Sudan and Syria. In response to the 2009 influenza pandemic, NAMRU-3 also worked closely with the WHO to train over 70 participants from 32 countries in North/West Africa, Central Asia, and the Middle East on real-time reverse transcriptase polymerase chain reaction (rRT-PCR) using the CDC H1N1 assay kits. In the Lao People’s Democratic Republic, initial cases of A/H1N1 were tested at the National Center for Laboratory and Epidemiology using equipment and supplies furnished by NAMRU-2 and AFHSC-GEIS funding. All of these efforts helped strengthen the global public health community.

## Research, innovation and integration

One of the primary focus areas within the strategic goal of research, innovation and integration is the development of rapidly deployable, field-expedient diagnostic platforms for influenza. Although they generally have a high specificity, point-of-care tests have a poor sensitivity for influenza, especially the 2009 novel A/H1N1 virus [16]. Moreover, while these tests can distinguish between influenza A and B viruses, they are rarely able to subtype specific viruses.

During 2009, AFHSC-GEIS supported the U.S. Army Medical Research Institute of Infectious Diseases (USAMRIID) and the Chemical Biological Medical Systems-Joint Program Management Office transition of the CDC H5N1 and novel H1N1 assays to the Joint Biological Agent Identification and Diagnostic System (JBAIDS). The system had been developed as a ruggedized PCR platform for field identification of priority pathogens of interest. During 2009, USAMRIID successfully optimized and tested the CDC H5N1 assays, and the data will be used in a DoD-sponsored 510K application to the Food and Drug Administration (FDA) for use on the JBAIDS in the future.

In addition, USAMRIID and NHRC successfully optimized the CDC H1N1 assay for use on the JBAIDS platform, and DoD submitted a request to the FDA to extend the current H1N1 Emergency Use Authorization for the JBAIDS H1N1 pandemic influenza assay. The FDA commissioner signed the request on Aug. 24, 2009. As a result, DoD now has the capability to provide a timely clinical diagnosis of novel A/H1N1 in U.S. servicemembers and civilians in deployed and field settings, and is well positioned to extend this capability to include H5N1 influenza diagnosis (Additional File 2).

In addition to the CDC H5N1 assays, AFHSC-GEIS also supported FDA approval of a rapid avian H5N1 influenza test (Arbor Vita Corp., AVantage™) using NHRC clinical trial data and validation of the National Veterinary Service Laboratory assay for the AI matrix, H5N1 and H7N3 strains on the JBAIDS by the DoD Veterinary Food Analysis and Diagnostic Laboratory at Fort Sam Houston, Texas. These capabilities will help to further increase DoD’s capacity for HPAI surveillance and outbreak response in remote settings.

In 2009, AFHSC-GEIS also sought to integrate influenza full genome sequencing within DoD. To this end, the Walter Reed Army Institute of Research (WRAIR) established full-length and ultra-deep, high-throughput genome sequencing. Twenty-six viruses (two seasonal A/H1N1, two A/H3N2, and 22 novel A/H1N1) from six countries were fully sequenced and submitted to GenBank. In turn, this sequencing provided valuable information on current viral mutations to DoD and the global public health community.

## Assessment and communication of value added

By utilizing data from the Defense Medical Surveillance System, AFHSC conducts assessments on influenza activity, safety of the novel A/H1N1 influenza vaccine and effectiveness of influenza vaccine. Influenza activity among all DoD beneficiaries is monitored weekly and summarized in a weekly report disseminated to service-specific public health centers, preventive medicine physicians and DoD leadership. In addition, AFHSC-GEIS generates a weekly summary of all influenza surveillance reports from DoD laboratories, service-specific public health centers, Combatant Commands and other AFHSC-GEIS partners. The weekly report is posted on the DoD Pandemic Influenza Watchboard (http://fhpr.osd.mil/aiWatchboard/). Both reports are valuable in providing DoD decision makers and global public health leaders with a timely and succinct accounting of influenza activity, severity and geographic distribution.

AFHSC has also partnered with the Military Vaccine Agency, CDC and FDA to provide weekly safety assessments of the novel A/H1N1 influenza vaccine among active component servicemembers. AFHSC provides the only data within DoD for this collaboration. As a result, the center plays a valuable role in the country’s assessment of the safety of this vaccine.

Each year, AFHSC-GEIS conducts mid-season assessments of the effectiveness of the seasonal vaccines, and during 2010, will examine the effectiveness of the novel A/H1N1 influenza vaccine. Initial estimates of seasonal vaccine effectiveness against novel A/H1N1-associated illness have been presented at scientific meetings and have been published. [17] Mid-season evaluations generated in January and February 2010 aimed to provide crucial information to the Vaccine and Related Biologic Products Advisory Committee and the public health community at large.

Additionally, network partners at NHRC and USAFSAM also evaluate vaccine effectiveness among important subpopulations throughout DoD. NHRC has established a framework for evaluating influenza vaccine effectiveness among basic military trainees that has served as a valuable tool in the larger effort to monitor this important indicator. USAFSAM works diligently each season to identify and molecularly analyze viruses from cases considered potential vaccine breakthroughs (e.g., cases occurring ≥14 days after vaccination) as determined by the surveillance questionnaire data collected as part of routine sentinel surveillance. The Defense Department is well positioned to determine the overall effectiveness of both seasonal and pandemic vaccines in military populations. However, results of these evaluations may not be generalizable to the population at large, given the young, healthy and highly vaccinated nature of military populations. This function is viewed favorably and of great value to the vaccine and public health communities.

## Discussion

Although many goals were accomplished during this past year, the novel A/H1N1 influenza virus pandemic of 2009 presented unique management challenges for AFHSC-GEIS and its network of partners. The first significant problem centered on “sensitivity” in terms of reporting cases to host-country health authorities, while simultaneously providing U.S. military and civilian health agencies with the reports. Although the identification of cases was important, many host-country officials perceived that reporting of cases could be detrimental to their economy or community. In tandem with the high visibility of reports, laboratory testing associated with the large increase in processing specimens presented a challenge in terms of local and regional expectation of timely results that each partner needed to address. The consolidation of testing results was found to be challenging, thus, the authors see a pressing need for standardization of reporting in the future. Other challenges occurred in terms of achieving effective ongoing strain-sequencing analysis and reporting to CDC and WHO officials. Lastly, given the high volume of testing required during a pandemic, the program must take a closer look at changing the paradigm of testing whereby only a representative portion (i.e., 10 percent to 20 percent), instead of every sample, is given priority. For example, researchers could test severe cases (e.g., SARI, hospitalized, pneumonia) to more effectively provide reliable estimates of the virus’ impact.

A primary focus of the AFHSC-GEIS influenza surveillance system in 2010 is the continued monitoring and tracking of novel A/H1N1 virus for changes in severity, antiviral resistance or transmissibility, particularly in our special populations (e.g., recruits, deployed and shipboard personnel) within the military. Sentinel-based surveillance by our partners (e.g., USAFSAM, LRMC, BAACH) will continue to remain a key component of the surveillance program, as will the population-based surveillance at eight of 10 military recruit-training centers and the Pacific Rim Surveillance at Naval Hospital Yokosuka by NHRC.

To expand DoD’s surveillance efforts within the Military Health System, AFHSC-GEIS is partnering with the Infectious Disease Clinical Research Program to establish an acute respiratory infection consortium at several large DoD medical treatment facilities. This group will examine the pandemic’s impact on the U.S. military health care population and evaluate the effectiveness of potential intervention measures, including vaccine-specific effectiveness and non-vaccine interventions, such as hand washing, febrile screening, cohorting and recruit space allocation.

Besides continued surveillance within DoD, AFHSC-GEIS partners are fostering and developing new relationships for surveillance within other military populations to expand the center’s global surveillance program and enhance its contribution to global public health. Examples of potential future collaborations include NMRCD partnerships with Bolivia and Ecuador; AFRIMS surveillance in Vietnam; NAMRU-3 development of a veterinary and human influenza surveillance network in western Africa with Burkina Faso, Cote d’Ivoire and Ghana armed forces; establishment of a central African military alliance by the Global Viral Forecasting Initiative in Cameroon; and expansion of DoD and foreign military influenza and EID surveillance efforts in East Africa (Kenya, Tanzania and Uganda) and Central America (El Salvador, Guatemala and Honduras). Further expansion of AFHSC-GEIS-sponsored partnerships with Ministries of Health will also be explored (e.g., by PHCR-South in Central America) to provide improved surveillance in regions of the world where surveillance is lacking or inadequate.

Surveillance of zoonotic influenza will become more focused in 2010. While waterfowl, especially ducks and geese, can be infected and shed many subtypes of influenza A viruses, viral presence in these species does not necessarily imply a risk to humans due to the potential for species-specific strains and especially a lack of human exposure to the waterfowl necessary for transmission [18]. As a result, instead of focusing on migratory birds, AFHSC-GEIS will concentrate on those areas, such as live-bird markets, abattoirs and large breeding farms, and occupations, such as backyard agricultural workers, where individuals are more likely to be exposed to animals, thus selecting a subset that is at much higher risk of infection with, and transmission of, zoonotic influenza strains.

Finally, AFHSC-GEIS will seek to expand DoD’s capability to analyze and characterize viruses in-house. In 2009, AFHSC-GEIS funding was used to equip several laboratories with antiviral resistance testing capacity (genotypically by pyrosequencing and phenotypically by inhibition in culture). The capability will be further developed in the future. Likewise, WRAIR’s genomic sequencing capacity and throughput will also increase. Additionally, funding will be provided to expand the JBAIDS influenza capability from novel A/H1N1 and H5N1 to include pan-influenza A, pan-influenza B, H1 seasonal and H3 seasonal viruses.

## Conclusions

 The 1996 Presidential Directive charged the U.S. DoD with monitoring EIDs in the military population. The directive led to creation of the AFHSC-GEIS influenza surveillance system, which strives to be a valuable asset to DoD and the global public health community. Its specimen catchment area includes regions noted for their regular contribution to global strain circulation, such as Southeast Asia, as well as South America, Africa and the Middle East, where strain circulation information is limited.

During 2009, the AFHSC-GEIS influenza and respiratory disease surveillance network not only detected the first cases of novel A/H1N1 among U.S. military beneficiaries, but also detected the first laboratory-confirmed cases within the United States and many countries throughout the world [19]. In addition to its EID surveillance, the network also assisted with providing a rapid global response to the 2009 influenza pandemic through training and capacity-building efforts with partner nations, developing new surveillance and diagnostic platforms, and timely reporting of surveillance results and disease trends to public health authorities such as the CDC and WHO.

This extensive network is positioned to detect the emergence of new respiratory pathogens or significant mutations in the novel A/H1N1 2009 influenza virus as they transpire. The projects and initiatives in 2010 and beyond will help to further strengthen and maintain this network, and ultimately contribute to the sustainment of force health protection and global public health.

## Competing interests

The authors declare that they have no competing interests.

## Authors' contributions

RLB was responsible for oversight and management of the AFHSC-GEIS Avian and Pandemic Influenza Department and drafted the manuscript. KGV, MCJ and JLS were responsible for regional oversight and management within the AFHSC-GEIS Avian and Pandemic Influenza Department. AAE was responsible for analysis of influenza trends within the U.S. DoD. JAP and RGP provided oversight and conducted AFHSC-GEIS respiratory disease surveillance at AFRIMS. JLM was responsible for program management of the CDHAM pandemic influenza training support to U.S. Combatant Commands. MQ provided oversight and conducted AFHSC-GEIS respiratory disease surveillance at PHCR-South. TP and MJC provided oversight and conducted AFHSC-GEIS respiratory disease surveillance at LRMC and PHCR-Europe. JG provided oversight and conducted AFHSC-GEIS respiratory disease surveillance at PHCR-Pacific. DS and JW provided oversight and conducted AFHSC-GEIS respiratory disease surveillance at USAMRU-K. AW and CCD were responsible for optimizing the avian H5 panel for use on the JBAIDS platform. MLB provided oversight and conducted AFHSC-GEIS respiratory disease surveillance at BAACH. ST, MRK and MW provided oversight and conducted AFHSC-GEIS respiratory disease surveillance at NAMRU-2. JAT and BO provided oversight and conducted AFHSC-GEIS respiratory disease surveillance at NAMRU-3. PJB and AH provided oversight and conducted AFHSC-GEIS respiratory disease surveillance at NHRC. JMM, HR and AL provided oversight and conducted AFHSC-GEIS respiratory disease surveillance at NMRCD. RJS and DAN were responsible for optimizing the H5N1 assay for use on the JBAIDS platform. VHM and TG provided oversight and conducted AFHSC-GEIS respiratory disease surveillance at USAFSAM. TS provided oversight and conducted AFHSC-GEIS respiratory disease surveillance at Navy Environmental and Preventive Medicine Unit-2. GCG provided oversight and conducted AFHSC-GEIS respiratory disease surveillance at the University of Iowa. DLB was responsible for oversight and management of AFHSC-GEIS operations and developed the reporting format. KLR was the director of DoD GEIS and provided oversight and direction of its surveillance activities.

## Supplementary Material

Additional File 1 AFHSC-GEIS supports pandemic H1N1 influenza outbreak response in Kenya and East Africa. Figure - Laboratory-confirmed cases of novel A/H1N1 influenza in Kenya, as of Oct. 8, 2009. Click here for file

Additional File 2 Deployment of the JBAIDS for diagnosis of novel A/H1N1 influenza in the deployed operations. Figure - Use of the JBAIDS for diagnosis of the novel A/H1N1 influenza virus in a deployed setting. Click here for file

## References

[B1] Clinton WJ Presidential Decision Directive NSTC-7 1996 The White House

[B2] Gray GC Acute respiratory disease in the military Federal Practioner 1995 12 27 33

[B3] Pazzaglia G Pasternack M Recent trends of pneumonia morbidity in U.S. Naval personnel Mil Med 1983 148 8 647 651 6415517

[B4] Gray GC Callahan JD Hawksworth AW Fisher CA Gaydos JC Respiratory diseases among U.S. military personnel: countering emerging threats Emerg Infect Dis 1999 5 3 379 385 10.3201/eid0503.990308 10341174 PMC2640764

[B5] Brundage JF Interactions between influenza and bacterial respiratory pathogens: implications for pandemic preparedness The Lancet infectious diseases 2006 6 5 303 312 10.1016/S1473-3099(06)70466-2 16631551 PMC7106411

[B6] Canas LC Lohman K Pavlin JA Endy T Singh DL Pandey P Shrestha MP Scott RM Russell KL Watts D The Department of Defense laboratory-based global influenza surveillance system Mil Med 2000 165 7 Suppl 2 52 56 10920641

[B7] Kelley PW A commentary on the military role in global influenza surveillance Am J Prev Med 2009 37 3 260 261 10.1016/j.amepre.2009.06.003 19666164

[B8] Owens AB Canas LC Russell KL Neville JS Pavlin JA MacIntosh VH Gray GC Gaydos JC Department of Defense Global Laboratory-Based Influenza Surveillance: 1998-2005 Am J Prev Med 2009 37 3 235 241 10.1016/j.amepre.2009.04.022 19666159

[B9] Sueker JJ Blazes DL Johns MC Blair PJ Sjoberg PA Tjaden JA Montgomery JM Pavlin JA Schnabel DC Eick AA Influenza and respiratory disease surveillance: the U.S. military's global laboratory-based network Influenza Other Respi Viruses 2010 4 3 155 161 10.1111/j.1750-2659.2010.00129.x 20409212 PMC4941663

[B10] Swine influenza A (H1N1) infection in two children—Southern California, March-April 2009 MMWR Morb Mortal Wkly Rep 2009 58 15 400 402 19390508

[B11] Update: swine influenza A (H1N1) infections—California and Texas, April 2009 MMWR Morb Mortal Wkly Rep 2009 58 16 435 437 19407739

[B12] Host range and emerging and reemerging pathogens http://www.ncbi.nlm.nih.gov/entrez/query.fcgi?cmd=Retrieve&db=PubMed&dopt=Citation&list_uids=16485468

[B13] Blair PJ Wierzba TF Touch S Vonthanak S Xu X Garten RJ Okomo-Adhiambo MA Klimov AI Kasper MR Putnam SD Influenza epidemiology and characterization of influenza viruses in patients seeking treatment for acute fever in Cambodia Epidemiol Infect 138 2 199 209 10.1017/S095026880999063X 19698213

[B14] Otto J Review: Training efforts within AFHSC/GEIS BMC Public Health in press

[B15] Sanchez JL Review: Capacity building within AFHSC/GEIS BMC Public Health in press 10.1186/1471-2458-11-S2-S4 PMC3092414 21388564

[B16] Vasoo S Stevens J Singh K Rapid antigen tests for diagnosis of pandemic (Swine) influenza A/H1N1 Clin Infect Dis 2009 49 7 1090 1093 10.1086/644743 19725784 PMC7107932

[B17] Johns MC Eick AA Blazes DL Lee SE Perdue CL Lipnick R Vest KG Russell KL DeFraites RF Sanchez JL Seasonal influenza vaccine and protection against pandemic (H1N1) 2009-associated illness among U.S. military personnel PloS one 5 5 e10722 10.1371/journal.pone.0010722 20502705 PMC2873284

[B18] Munster VJ Fouchier RA Avian influenza virus: of virus and bird ecology Vaccine 2009 27 45 6340 6344 10.1016/j.vaccine.2009.02.082 19840670

[B19] Dawood FS Jain S Finelli L Shaw MW Lindstrom S Garten RJ Gubareva LV Xu X Bridges CB Uyeki TM Emergence of a novel swine-origin influenza A (H1N1) virus in humans The New England Journal of Medicine 2009 360 25 2605 2615 10.1056/NEJMoa0903810 19423869

