# Supplementary material for: Department of Defense influenza and other respiratory disease surveillance during the 2009 pandemic
Source: BMC Public Health. 2011 Mar 4;11(Suppl 2):S6. doi: 10.1186/1471-2458-11-S2-S6 (PMC3092416; doi:10.1186/1471-2458-11-S2-S6)

In response to the 2009 pandemic H1N1 influenza outbreak, the Kenya Ministry of Public Health and Sanitation (MoPHS) began implementing its influenza pandemic preparedness plans in mid May. The MoPHS consulted with officials from the AFHSC-GEIS-funded U.S. Army Medical Research Unit-Kenya (USAMRU-K) laboratory on preparedness, diagnostic and public outreach planning prior to implementing the plan.

On June 29, 2009, the Centers for Disease Control and Prevention-Kenya (CDC-Kenya) and the AFHSC-GEIS influenza program at USAMRU-K jointly confirmed the first case of novel A/H1N1 in Kenya. Working with the Kenya MoPHS, personnel from the World Health Organization (WHO), CDC-Kenya and USAMRU-K developed case definitions, hospital response programs, risk communications messages, and laboratory testing algorithms for the pandemic. USAMRU-K also assisted with the deployment of medical education training teams that traveled around the country to train 756 healthcare providers on matters relating to the pandemic H1N1 influenza outbreak. In tandem with its mandate as a regional laboratory, the Kenya National Influenza Centre (NIC), with the assistance of USAMRU-K, also served as the reference and diagnostic laboratory for pandemic influenza diagnosis for the Republic of Seychelles and Republic of Somalia.

Within three months of its introduction into Kenya, the novel A/H1N1 influenza virus had spread to many regions within the country (Vignette Figure 1). In the first three months of the pandemic outbreak, 1,439 samples were sent to the NIC for diagnostic testing and 233 samples (16 percent) tested positive for the novel A/H1N1 influenza.

From May 1 to Oct. 10, the Kenya NIC received 99 samples from suspected cases of novel A/H1N1 influenza in the Republic of Seychelles. Nearly half (46 percent) of the specimens were positive for influenza. Among positive influenza cases, a majority (76 percent) was novel A/H1N1. During this same period, the Kenya NIC received 10 samples from the Garowe Hospital in Somalia. Two specimens tested positive for the novel A/H1N1 virus and four tested positive for seasonal influenza A.

By leveraging the previously developed capacity built in support of human influenza sentinel surveillance, USAMRU-K and AFHSC-GEIS supported the rapid diagnosis and response to the 2009 H1N1 influenza pandemic within the region, consistent with its global mandate and objectives.


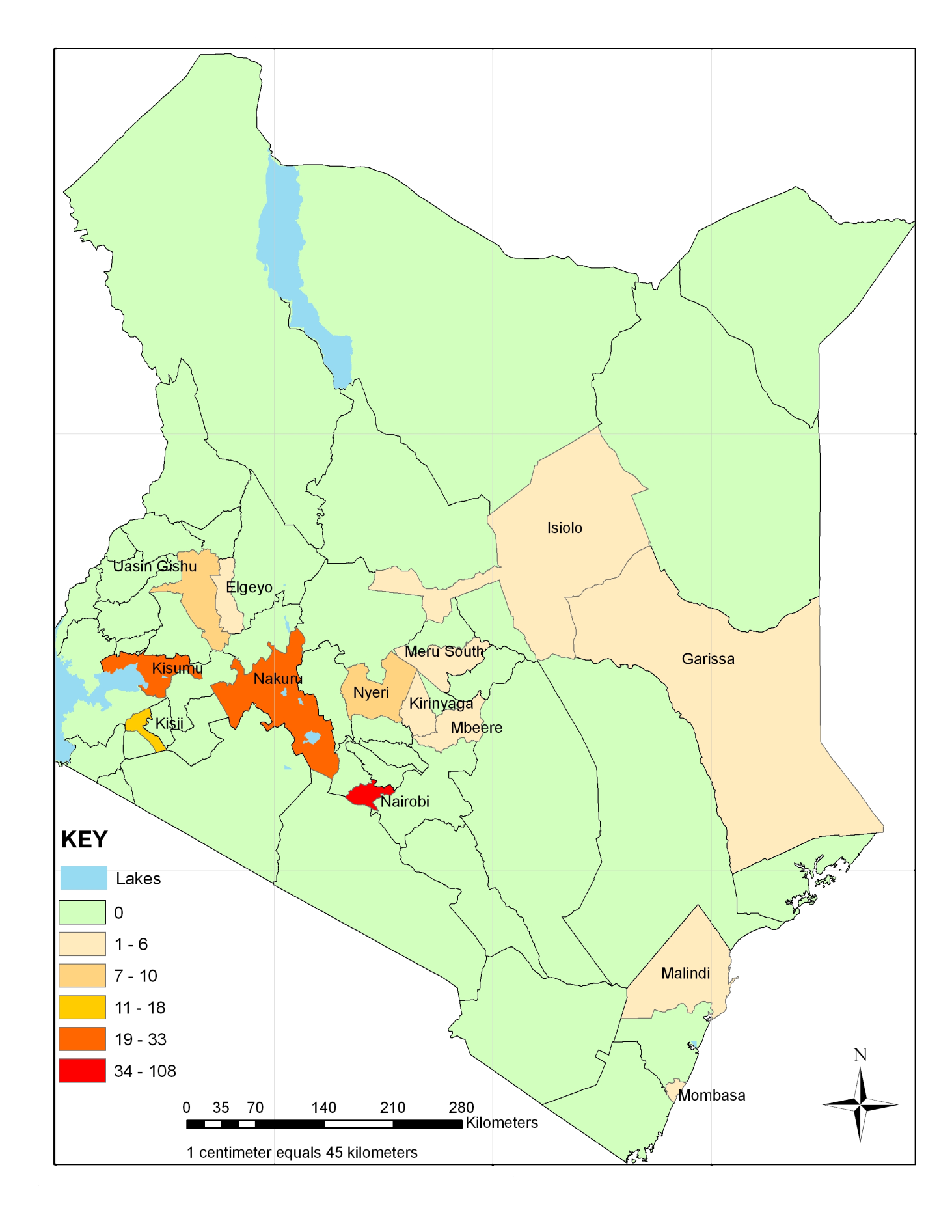

Supplement: Additional File 1 — AFHSC-GEIS supports pandemic H1N1 influenza outbreak response in Kenya and East Africa. Figure - Laboratory-confirmed cases of novel A/H1N1 influenza in Kenya, as of Oct. 8, 2009. [file 1471-2458-11-S2-S6-S1.docx]
