# Supplementary material for: Department of Defense influenza and other respiratory disease surveillance during the 2009 pandemic
Source: BMC Public Health. 2011 Mar 4;11(Suppl 2):S6. doi: 10.1186/1471-2458-11-S2-S6 (PMC3092416; doi:10.1186/1471-2458-11-S2-S6)
Supplement: Additional File 2 — Deployment of the JBAIDS for diagnosis of novel A/H1N1 influenza in the deployed operations. Figure - Use of the JBAIDS for diagnosis of the novel A/H1N1 influenza virus in a deployed setting. [file 1471-2458-11-S2-S6-S2.doc]

The battle against the A/H1N1 influenza virus has taken a turn for the better with the introduction of the Joint Biological Agent Identification and Diagnostic System (JBAIDS) in field laboratories within the U.S. Central Command region. With the support of the Armed Forces Health Surveillance Center-Global Emerging Infections and Surveillance and Response System (AFHSC-GEIS), Navy Medical Research Unit No. 3 (NAMRU-3) and Chemical Biological Medical Systems-Joint Program Management Office (CBMS-JPMO) provided training and capability for real-time diagnosis of novel A/H1N1diagnostic in the deployed setting.

To confirm influenza cases, JBAIDS was employed as a stand-in for the Applied Bio-systems (ABI) 7500 FAST real**-**time polymerase chain reaction (PCR) system, a more sensitive instrument that is not yet readily available in combat zones. The extension of the Food and Drug Administration’s Emergency Use Authorization in August 2009 to include use of the Centers for Disease Control and Prevention H1N1 real-time PCR diagnostic kit, allowed NAMRU-3 researchers to be certified and serve as regional JBAIDS trainers for real-time detection of the pandemic H1N1influenza virus of 2009.

On short notice, NAMRU-3 was able to deploy a team of four personnel to train and certify 15 laboratory medical personnel on the JBAIDS platform at five installations in Central Command. Following completion of training and certification on JBAIDS, combat field clinics now possess the means to clinically diagnose novel A/H1N1 influenza in patients presenting with influenza-like illnesses.

Because of its versatility and portability, JBAIDS promises to offer a significant advantage in the combat zone. Continued collaborations among AFHSC-GEIS, NAMRU-3 and CBMS-JPMO will ensure that JBAIDS usage thrives on and off the battlefield.
